# Effects of BmCPV Infection on Silkworm *Bombyx mori* Intestinal Bacteria

**DOI:** 10.1371/journal.pone.0146313

**Published:** 2016-01-08

**Authors:** Zhenli Sun, Yahong Lu, Hao Zhang, Dhiraj Kumar, Bo Liu, Yongchang Gong, Min Zhu, Liyuan Zhu, Zi Liang, Sulan Kuang, Fei Chen, Xiaolong Hu, Guangli Cao, Renyu Xue, Chengliang Gong

**Affiliations:** 1 School of Biology & Basic Medical Science, Soochow University, Suzhou, 215123, China; 2 National Engineering Laboratory for Modern Silk, Soochow University, Suzhou, 215123, China; Institute of Plant Physiology and Ecology, CHINA

## Abstract

The gut microbiota has a crucial role in the growth, development and environmental adaptation in the host insect. The objective of our work was to investigate the microbiota of the healthy silkworm *Bombyx mori* gut and changes after the infection of *B*. *mori* cypovirus (BmCPV). Intestinal contents of the infected and healthy larvae of *B*. *mori* of fifth instar were collected at 24, 72 and 144 h post infection with BmCPV. The gut bacteria were analyzed by pyrosequencing of the 16S rRNA gene. 147(135) and 113(103) genera were found in the gut content of the healthy control female (male) larvae and BmCPV-infected female (male) larvae, respectively. In general, the microbial communities in the gut content of healthy larvae were dominated by *Enterococcus*, *Delftia*, *Pelomonas*, *Ralstonia* and *Staphylococcus*, however the abundance change of each genus was depended on the developmental stage and gender. Microbial diversity reached minimum at 144 h of fifth instar larvae. The abundance of *Enterococcus* in the females was substantially lower and the abundance of *Delftia*, *Aurantimonas* and *Staphylococcus* was substantially higher compared to the males. Bacterial diversity in the intestinal contents decreased after post infection with BmCPV, whereas the abundance of both *Enterococcus* and *Staphylococcus* which belongs to Gram-positive were increased. Therefore, our findings suggested that observed changes in relative abundance was related to the immune response of silkworm to BmCPV infection. Relevance analysis of plenty of the predominant genera showed the abundance of the *Enterococcus* genus was in negative correlation with the abundance of the most predominant genera. These results provided insight into the relationship between the gut microbiota and development of the BmCPV-infected silkworm.

## Introduction

The silkworm *Bombyx mori* is an economically important domesticated insect considered as an ideal model organism for Lepidoptera. The yield and quality of cocoons depended on the silkworm strain, climatic condition, silkworm health and absorption of nutrients. Digestive absorption, nutrient utilization and diseases emergence of the silkworm was closely related to microbiota found in the midgut of the silkworm larvae [1]. Therefore, knowledge of the dynamic change of gut bacteria post infection should lead to the improvement of the health and nutrient absorption of the silkworm.

The dilution culture method of intestinal juice was used to investigate the microbiota in the silkworm gut at different developmental stages, 253 strains of bacteria belonging to 16 genera were isolated[2]. In another study, 89 *Enterococcus spp*. isolates were detected in the intestinal content of the healthy silkworm larvae and adult moths using the API 20 STREP (V.5.0) system (BIOMERIEUXS. A., France) based on numerical taxonomy [3]. Usually, only a few predominant bacterial genera can be isolated by the culture-dependent method; bacterial genera with low abundance tend not to be found with the culture-dependent method. The 16S rRNA gene, common to all prokaryotes, was often used as a marker for identifying bacterial species [4]. Using of the 16S rRNA gene has been a powerful tool for the detection and authentication of bacteria. Further, restriction pattern of the 16S rRNA gene amplified from the gut bacterial metagenomic DNA of silkworm, can be used to detect the bacteria population belonging to the *Arthrobacter*, *Lactobacillus*, *Pseudomonas*, *Escherichia*, *Micrococcus*, *Bacillus* and *Staphylococcus* genera [1]. Although more additional bacteria can be identified by traditional molecular biology methods compared to the culture-dependent method, diversity and richness of gut microbiota may be underestimated by traditional molecular biology methods.

The silkworm gut microbiota was impacted by forages, four predominant genera (*Brevundimonas*, *Stenotrophomonas*, *Enterobacter* and *Staphylococcus*) were shared by silkworms cultivated on leaves of *Cudrania tricuspidata* or mulberry (*Morus* spp.). Five additional abundant genera (*Aeromonas*, *Brevibacterium*, *Citrobacter*, *Escherichia* and *Klebsiella*) and two additional abundant genera (*Pseudomonas* and *Agrobacterium*) were recorded from the gut microbiota of silkworm larvae cultivated on mulberry and *C*. *tricuspidata* leaves respectively [5]. The distribution of intestinal bacteria was also changed with regard to the state of health of the silkworm; the number of bacterial species of the genus *Enterococci* decreased, while the number of bacteria of the genus *Enterococci* was increased in silkworms infected with *Nosema bombycis* which caused the epidemic disease pebrine compared to healthy silkworms [6].

Bacterial intestinal diseases of *B*. *mori* can be caused by abnormal multiplication of bacteria in the gut; *B*. *mori* cypovirus (BmCPV) specially infected the epithelial cells of the silkworm midgut, silkworm cytoplasmic polyhedrosis caused by infection with BmCPV usually accompanies silkworm bacterial intestinal disease. To understand the effect of BmCPV infection on silkworm gut microbiota, in the present study, we explored the difference of microbiota between healthy female and male silkworm larvae and the changes of bacterial diversity after infection with BmCPV using pyrosequencing of the 16S rRNA gene. The results indicated that 147 and 135 genera were detected in the gut of healthy female and male silkworm larvae, respectively. The diversity of bacterial microbiota was reduced post infection with BmCPV; only 113 and 103 genera were observed in the female and male silkworm larvae, respectively. These results provided insight into the relationship between the gut microbiota and development of the BmCPV-infected silkworm.

## Materials and Methods

### Collection of intestinal contents

*B*. *mori* larvae of Daizo strain were cultured at 25°C on mulberry leaves and at a suitable humidity of about 70% ±5% together with a photoperiod of 14 h of light and 10 h of dark. Polyhedra were extracted from silkworm intestine which have infected with BmCPV, it has purified by multi-layer gauze and centrifuged using 6000r/min and then resuspended in 1×PBS to 10^8^ polyhedra ml^-1^. The newly molted fifth instar larvae were fed for 8 h on leaves smeared with BmCPV (10^8^ polyhedra ml^-1^) and then fed on untreated leaves. The midgut of 30 larvae (15 females and 15 males) was dissected out in a sterile environment at 24, 72 and 144 h post infection, respectively. The collected intestinal contents were immediately frozen and stored at –80°C. Silkworms fed for 8 h on mulberry leaves smeared with sterilized double distilled water were used as control.

### DNA extraction, amplification, purification and sequencing

Total genomic DNA was extracted from the intestinal contents using the Z.E.N.A Soil DNA Kit (Omega Bio-Tek, GA, D5625-01). The quality of the extracted DNA was assessed by electrophoresis in 1% (w/v) agarose gel. The concentration of the extracted DNAs was determined using a Qubit® 2.0 Fluorometer (Life Technologies, California USA) and then normalized to 10 ng μl^-1^. Universal 16S rRNA genes were amplified by PCR in a volume of 50 μl containing 10 ng DNA, 5 μl 10 × PCR buffer, 0.5 μl each dNTP (10 mM), 0.5 μl Plantium Taq (5 U μl^-1^), 0.5 μl bar-primers (50 μM) V1F:(5ʹ-*CGTATCGCCTCCCTCGCGCCATCAG*(barcode)AGAGTTTGATCMTGGCTCAG-3ʹ) and V3R2: (5ʹ-*CTATGCGCCTTGCCAGCCCGCTCAG*GTATTACCGCGGCTGCTGGCAC-3ʹ) modified at the 5^ʹ^ end to contain the 454 FLX Titanium Lib L adapters B (italics and underlined) and A (italics and underlined), respectively. The forward primers also contained a six base barcode sequence located between the primer sequence and the adapter. A unique barcode was used for each sample [7]. The thermocycling protocol was as follows: 94°C for 3 min, then 5 cycles of 94°C for 30 s, 45°C for 20 s, 65°C for 30 s, followed by 20 cycles of 94°C for 20 s, 55°C for 20 s, 72°C for 30 s, and a final extension step at 72°C for 5 min. Amplicons were purified using PCR purification kit (Sangon, Shanghai, China), and quantified by Qubit^®^ 2.0 fluorometer (Life Technologies, California, USA) and then pooled for 454 pyrosequencing by the Encode Genomics Bio-Technology Co., Ltd., Suzhou, China.

### Analysis of sequence data

Initially, the SFF file output from the sequencer was converted into fasta and qual files using the sffinfo program included in the 454 Life Sciences software package (Roche Diagnostics, Basel). Samples were distinguished by the barcode sequences and de-multiplexed reads were processed using LUCY (version 1.2) [8] to filter out reads with low-quality segments. Each valid read was had the following criteria; (1) contain a primer sequence ≥50 bp long; (2) contain no ambiguous base; (3) match the primer; and (4) be one of the used barcode sequences. Unique sequences were clustered into operational taxonomic units (OTUs) defined at the 97% similarity threshold. Taxonomical classification of the OTU-representative reads down to the genus level was carried out using Mothur’s version of the Ribosomal Database Project (RDP) Bayesian classifier through a normalized RDP training dataset [9]. The relative abundances of individual OTUs in a given assembly were estimated as the percentage of each individual OTU DNA relative to the sum of the total amplified DNA. Alpha diversity analysis was performed by Mothur software [10]. Rarefaction curves were used to assess species richness [11]. The Shannon–Wiener and Simpson diversity indexes were adopted to evaluate the bacterial diversity [12]. Chao1 [13] and Ace [14] indexes were used to estimate the total number of species in samples. The OTUs were aligned through PyNAST with a minimum alignment length of 150 bp and a minimum identity of 75% [15]. After alignment, PH LANE mask (http://greengenes.lbl.gov/) was used to screen away the hypervariable regions.

### Relevance analysis between samples

The phylogenetic tree was inferred using the neighbor-joining method with MEGA6.0 [16, 17]. Principal coordinate analyses (PCoA) were carried out using R software version 2.10.0(R Development Core Team, 2009, http://www.r-project.org) to compare bacterial community structures based on weighted-UniFrac from each library. The weighted-UniFrac distances were subjected to analysis of molecular variance (AMOVA) in Mothur to compare significant differences between bacterial communities from each sample [18, 10]. Principal components analysis (PCA) with linear ordination methods was utilized to explore the correlation between dominant genera using CANOCO 4.5 according to ter Braakand Šmilauer [13]. A histogram was created using SPSS19.0 version on the basis of genera distributed among samples [14].Venn diagram curves were created with the online tool Venny (http://bioinformatics.psb.ugent.be/webtools/Venn/).

## Results

### Analysis of the pyrosequencing-derived dataset

**Fig 1:**
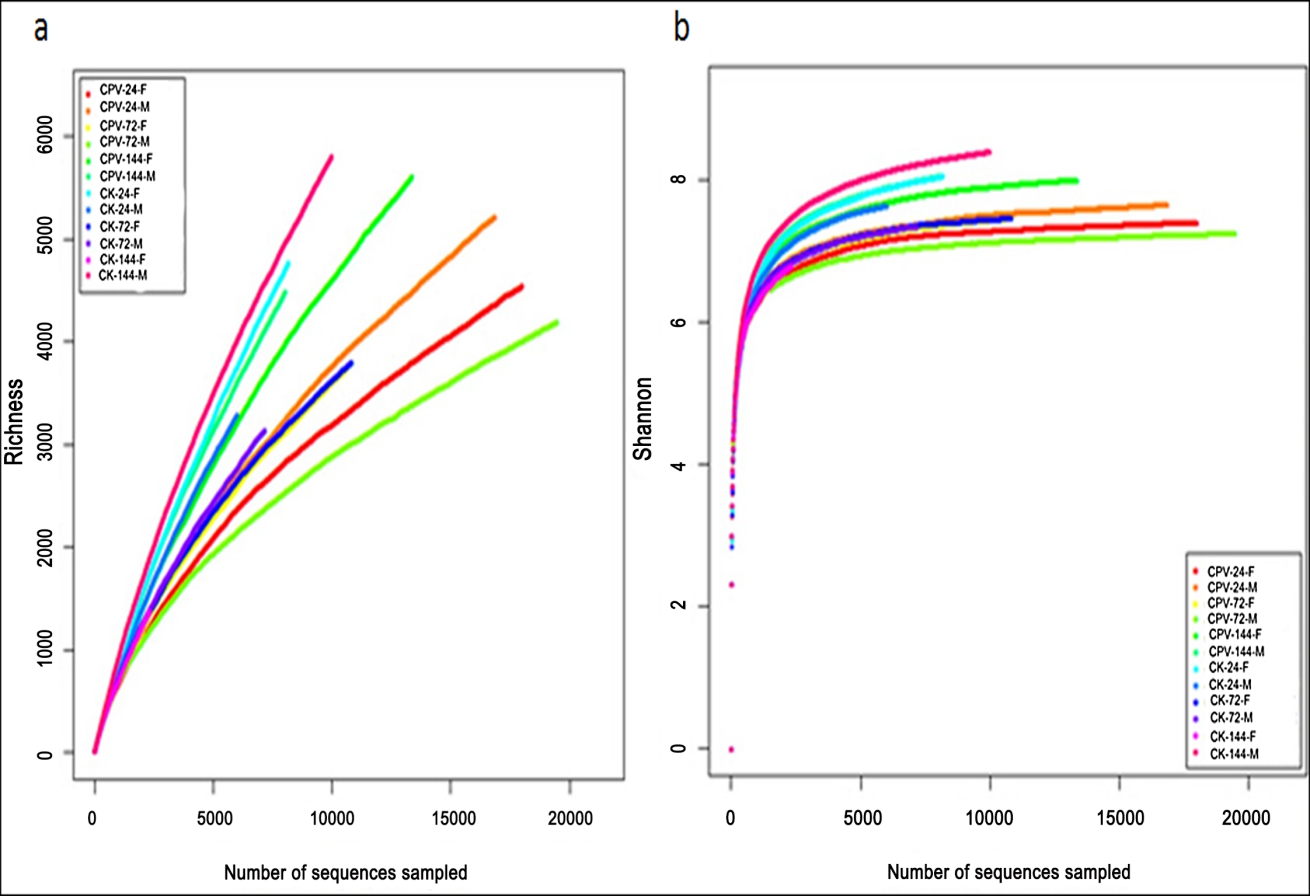
Richness rarefaction and Shannon index analysis of the different samples. CK (CPV)-24(72,144)-F (M) are samples mentioned in Table 1. (a) Rarefaction curves of OTUs clustered at 97% sequence identity across difference samples. (b) Rarefaction curves of the Shannon index according to OTU. Shannon indices approached a plateau and the rarefaction curves showed it ranged from 6 to 9.

After removal of low-quality reads, 60,551 valid reads were obtained from 12 samples using 454 pyrosequencing of the 16S rRNA gene. The total reads were 61,296,879 bp long, each ranging from 50 to 1150 bp with average of 468.9 bp. The number of reads differed for different samples; the number of reads for the 12 samples ranged from 1075 to 13,312. A total of 71.13% (43,072) of the total valid reads was assigned to a genus and 21,020 OTUs were obtained (Table 1). The richness/rarefaction curves for individual samples showed bacterial richness in the gut contents was different among 12 samples, although the rarefaction curves did not tend to approach the saturation plateau (Fig 1A), indicating the true bacterial richness in the silkworm gut was underestimated. Bacterial diversity was estimated by the Shannon index; the curves tended to plateau (Fig 1B) and the Shannon index of the gut microbiota of healthy silkworms was higher compared to BmCPV-infected silkworms, showing bacterial species diversity in the healthy silkworm gut was greater when compared with the BmCPV-infected silkworm gut and a similar result was obtained by Simpson index analysis. The OTU number of a bacterial community was estimated by Chao1 and Ace, the results indicated that community richness of silkworm gut depended on the developmental stage, gender and state of health, community richness of BmCPV-infected silkworm was greater compared to healthy silkworm (Table 2).

**Table 1:** Bacterial community in the contents of the silkworm gut.

Samples	valid reads	Reads assigned to genus	Phylum	Class	Order	Family	Genus	OTUs
CK-24-M	4377	2308	11	17	28	55	95	2479
CK-24-F	7084	5117	10	14	26	57	93	4111
CK-72-M	2880	1395	8	13	22	52	83	1532
CK-72-F	3226	1365	12	15	26	55	81	1637
CK-144-M	8979	7696	8	9	16	29	41	5116
CK-144-F	1872	922	5	11	18	39	70	1036
Total CK	28418	18803	16	29	40	94	199	12335
CPV-24-M	5511	3218	11	17	23	54	91	2398
CPV-24-F	2157	791	10	19	26	51	74	1363
CPV-72-M	1075	303	6	9	17	28	39	863
CPV-72-F	2146	866	8	12	22	43	72	1361
CPV-144-M	7932	7011	3	3	5	6	7	4400
CPV-144-F	13312	12080	5	18	12	19	28	5564
Total-CPV	32133	24269	14	26	35	77	156	12764

CK-24-F (M), CK-72-F (M) and CK-144-F (M) samples collected from the midgut contents of the fifth instar female (male) silkworm at 24, 72 and 144 h post feed with leaves smeared with water

CPV-24-F (M), CPV-72-F (M) and CPV-144-F (M) samples collected from midgut contents of the fifth instar female (male) silkworm at 24, 72 and 144 h post infection with BmCPV, the data for Chloroplast are omitted.

**Table 2:** Richness and diversity index in all samples.

Samples	Chao1	Shannon	Ace	Simpson
CK-24-F	1119.85	5.74	1187.01	0.91
CK-72-F	1223.89	2.82	1399.22	0.45
CK-144-F	745.64	5.68	879.45	0.91
CK-24-M	1085.98	6.05	1257.32	0.89
CK-72-M	1328.16	3.38	1441.29	0.55
CK-144-M	644.31	3.60	757.17	0.73
CPV-24-F	1207.82	1.92	1394.65	0.29
CPV-72-F	1251.55	2.45	1436.79	0.38
CPV-144-F	255.59	1.69	370.33	0.37
CPV-24-M	1193.34	3.05	1311.04	0.47
CPV-72-M	1053.20	1.58	1157.05	0.24
CPV-144-M	164.0	1.69	165.70	0.46

CK (CPV)-24(72,144)-F (M) are samples mentioned in Table 1. Chao1 and ACE indexes estimated the total number of species in samples. The Shannon–Wiener diversity index was used to assess bacterial diversity and its value positive correlation with microbial diversity.Simpson index was used to quantify the biodiversity of a habitat, its value negative correlation with microbial diversity.

### Composition of gut microbiota of the healthy silkworm

A total of 28,418 valid reads and 12335 OTUs were obtained after filtering out reads with low-quality segments. Because there are many chloroplast of mulberry in the intestinal contents of silkworm, the OTU-representative reads were assigned to phylum, class, order, family and genus using the RDP classifier [19] except reads representing chloroplast; 16 phyla, 29 classes, 40 orders, 94 families and 199 genera were detected in the intestinal contents of healthy fifth instar silkworm larvae (Table 1). Where 16 phyla namely Firmicutes, Proteobacteria, Actinobacteria, Bacteroidetes, Armatimonadetes, TM7, Thermotogae, Acidobacteria, OP11, Nitrospira, Gemmatimonadetes, Planctomycetes, Chloroflexi, Deinococcus-Thermus, Verrucomicrobia and Chlorobi were recorded, of which three most abundant phyla were Firmicutes (58.92%), Proteobacteria (39.41%) and Actinobacteria (1.28%).

The predominant genera in the gut contents of healthy silkworm larvae were determined to understand the other important bacteria. The predominant genera (>1%) were sequences related to *Enterococcus* (37.40%), *Delftia* (8.35%), *Pelomonas* (3.38%), *Ralstonia* (2.42%), *Tepidimonas* (1.97%), *Aurantimonas* (1.86%), *Pseudomonas* (1.63%), *Aspromonas* (1.56%) and *Staphylococcus* (1.06%).

### Changes of intestinal microbiota of the healthy silkworm during the growth period

**Fig 2:**
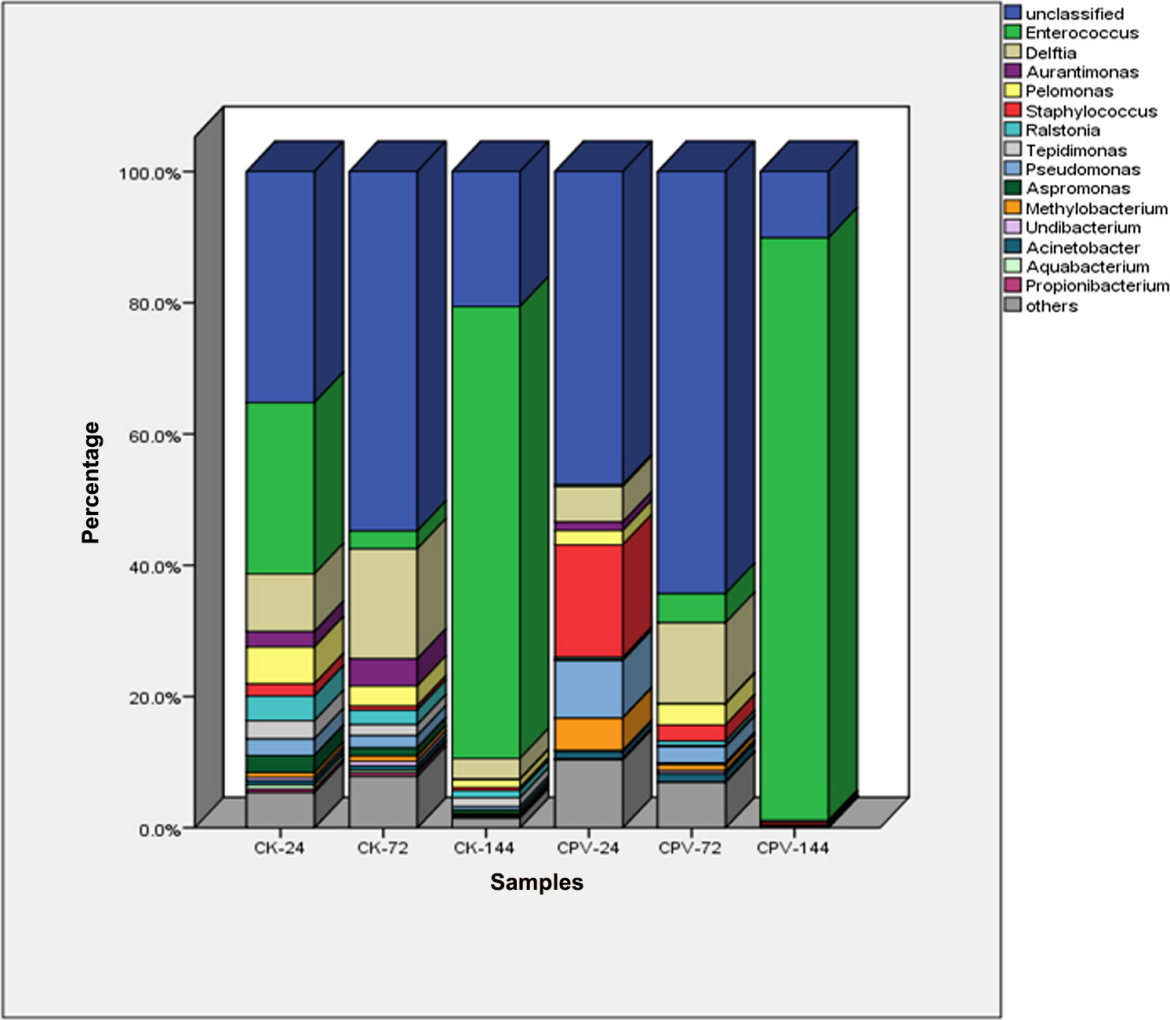
The dominant genera percentage for all samples at each time point. CK (CPV)-24(72,144)-F(M) are samples mentioned in Table 1. Relative read percentage of different bacterial genera within the different communities. Sequences that could not be classified into any known group were assigned as ‘Unclassified’

To understand the effect of developmental stages of silkworm on the intestinal microbiota, the changes of composition and abundance of bacteria in gut contents of healthy silkworm were determined according to the OTU-representative reads. At phylum level, the abundance of Proteobacteria and Actinobacteria decreased during the growth period of the fifth instar, especially from 72 to 144 h; other bacteria phyla accounted for smaller percentages in samples and displayed no regular pattern of change in fifth instar larvae. At genus level, differences in the composition of the bacterial community in the gut were also found at different developmental stages in the fifth instar. The proportions of some genera at different time points are given in S1 Table. Overall, the proportions of *Pelomonas*, *Ralstonia*, *Tepidimonas*, *Pseudomonas*, *Aspromonas*, *Staphylococcus* and *Aquabacterium* decreased with development of the silkworm. The abundance values of *Methylobacterium*, *Acinetobacter*, *Undibacterium* and *Propionibacterium* at 24 h of the fifth instar larvae were similar to those at 72 h and decreased at 144 h. The abundance of *Enterococcus* was the lowest at 72 h of the fifth instar larvae and the highest at 144 h. The abundance of *Delftia* increased with maturity, reached highest at 72 h of the fifth instar larvae and decreased at 144 h (Fig 2).

### Difference in the gut microbiota of the healthy silkworm between the male and female

At phylum level, the abundance of Actinobacteria in male larvae were substantially similar to female larvae at both 24 and 72 h of the fifth instar, whereas the abundance was decreased in both male and female larvae, and the abundance in females was 4.24 times higher than in males. Obvious difference in the richness of Proteobacteria was not found between male and female larvae either at 24 or 72 h of the fifth instar. The abundance in females was 3.17 folds higher than in males. The abundance of Firmicutes decreased in female larvae during development but increased in male larvae.

**Fig 3:**
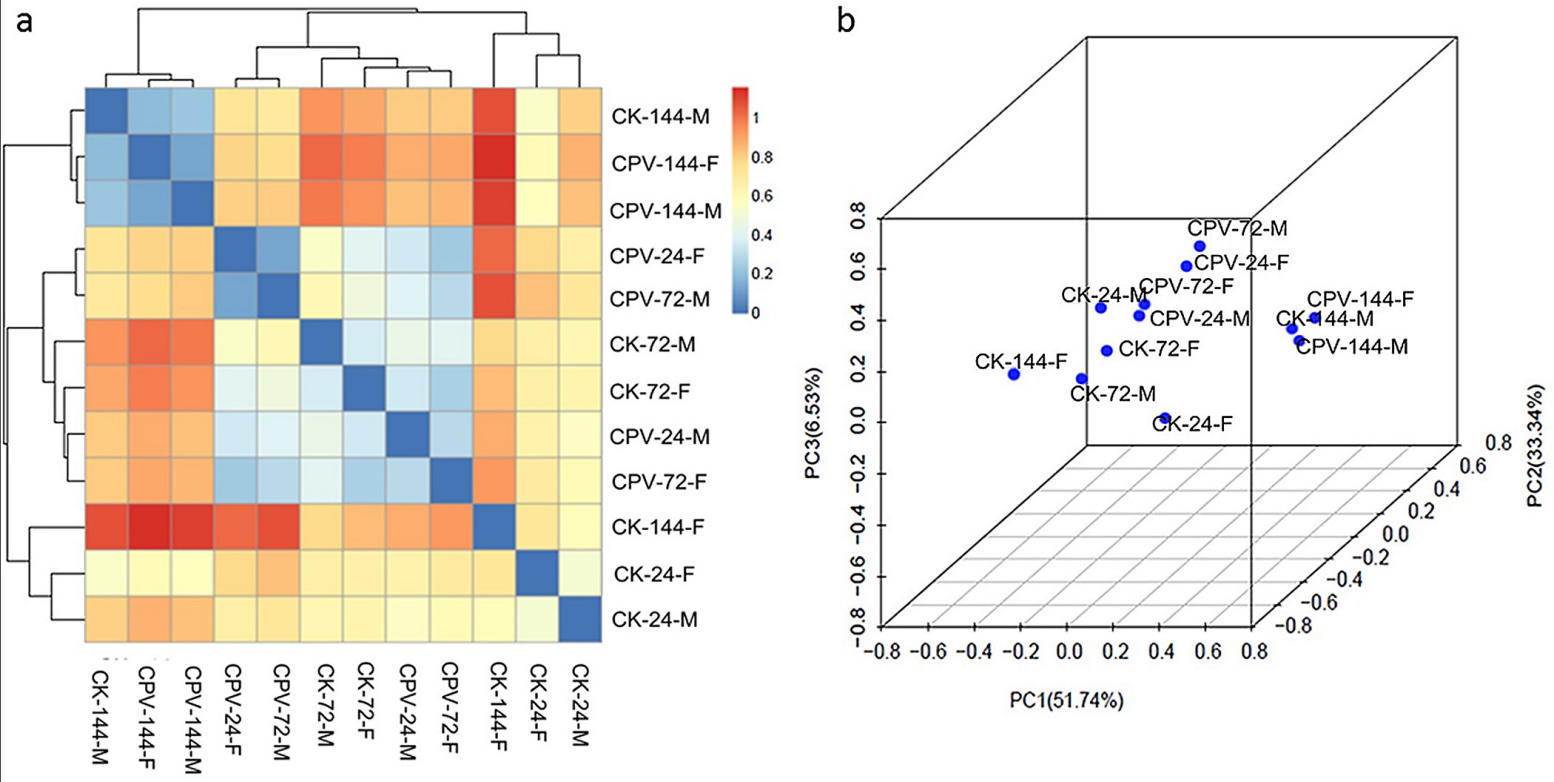
Heatmap and sorting analysis of the different samples. (a) Heatmap based on the hierarchical clustering solution (Bray–Curtis distance metric and complete clustering method) of the 12 samples. Rows and columns all represent the 12 samples, the similarity represented by the values in the heatmap and the heatmap of bacterial microbiota in different samples, unweighted UniFrac distance metric between samples using Unifrac was calculated to make the heatmap, the lower number represents greater similarity in bacterial microbiota between samples in the heatmap. (b) Sample sorting analysis, A PCoA plot was used to visualize the data based on β-diversity metrics of weighted UniFrac. Scatterplot of PCA-score depicting variance of fingerprints derived from different bacterial community. Principal components (PCs) 1, 2 and 3 explained 51.74%, 33.34% and 6.53% of the variance, respectively.

The gut microbiota in the larvae contained 147 genera in females and 135 in males,64 genera were found only in females and 52 found only in males; 83 genera were recorded in both genders (Fig 3). The abundance of predominant bacterial genera in the female (male) larvae was: *Enterococcus*, 24.75% (46.89%); *Delftia*, 12.57% (5.19%); *Pelomonas*, 3.27% (3.46%); *Ralstonia*, 2.21% (2.58%); *Tepidimonas*, 2.13% (1.85%); *Aspromonas*, 1.39% (1.69%); *Pseudomonas*, 1.61% (1.64%); *Aurantimonas*, 2.75% (1.20%); *Staphylococcus*, 1.87% (0.44%); *Acinetobacter* 0.68% (0.44%); and *Methylobacterium*, 0.48% (0.43%). In general, the abundance of *Enterococcus* in females was substantially lower when compared with males, and the abundance of *Delftia*, *Aurantimonas* and *Staphylococcus* were substantially higher.

### Change of intestinal microbiota after infection with BmCPV

BmCPV specially infects the epithelial cells of the silkworm midgut, and as the disease progresses, white wrinkles typically occurred in the posterior part of the midgut, and consequently, the digestive and absorptive functions of the midgut severely affected. To estimate the effect of BmCPV infection on gut microbiota, the change of gut microbiota after infection with the BmCPV was investigated. There were marked differences in the composition of the intestinal microbiota in BmCPV-infected silkworms compared to control healthy silkworms. 14 phyla, 26 classes, 35 orders, 77 families and 156 genera of bacteria were detected in the intestinal contents of BmCPV-infected silkworms, fewer compared to the healthy silkworm at all levels of classification (Table 1), suggesting bacterial diversity in the intestinal contents decreased post infection with BmCPV. The three most abundant phyla in BmCPV-infected larvae were Firmicutes (85.76%), Proteobacteria (13.13%) and Actinobacteria (0.91%) recorded, and the abundance of Firmicutes was increased by 45.55%, whereas abundances of Proteobacteria and Actinobacteria were respectively decreased by 66.68% and 28.91% compared to the healthy silkworm. In genus level, the predominant (>1%) genera post infection were *Enterococcus* (59.18%), *Staphylococcus* (4.58%), *Delftia* (2.70%), *Pseudomonas* (2.34%) and *Methylobacterium* (1.28%), roughly, the abundances of *Enterococcus* and *Staphylococcus* were increased and the abundance of *Delftia* was decreased after infection with the BmCPV.

The proportion of bacteria in the intestinal contents changed with the time course of BmCPV infection and the pattern of change depended on the type of bacteria present. The proportion of *Enterococcus* spp. decreased substantially at 24 h post infection with BmCPV and then increased to 88.75% at 144 h, which was a 1.28-fold increase compared to the control. The abundance of *Pseudomonas* spp. at 24 h post infection was 2.47-fold higher compared to the control and then decreased with the time course of BmCPV infection, reaching 0.02% at 144 h, which was 27-fold lower compared to the control. The abundance of *Staphylococcus* spp. at 24 h post infection was 17.16%, which was 9.28-fold greater than control, and then decreased to 0.37% at 144 h, which was similar to the control. The abundance of *Delftia* spp. decreased with the time course of BmCPV infection; the abundance at 24, 72 and 144 h post infection was lower compared to the control (S1 Table).

The change of bacterial microbiota in the BmCPV-infected female silkworms were different compared to the male; 113 genera were found in the females and 103 in the males, 53 unique genera were found in the females and 43 in the males, and 60 genera were found in both genders. The diversity of intestinal bacterial microbiota in BmCPV-infected female and male silkworm larvae decreased compared to the control. At 144 h post infection, the number of genera detected in the gut contents decreased sharply; only 28 genera were detected in females and 7 in males, compared to 70 and 41, respectively of control (Fig 2).

Post infection with BmCPV, roughly, the abundance of *Delftia*, *Pelomonas*, *Ralstonia*, *Tepidimonas*, *Aspromonas* and *Aurantimonas* genera decreased in both genders. The abundance of both *Enterococcus* and *Staphylococcus* increased in females but there was no significant change in males. Post infection with BmCPV, the richness of *Pseudomonas* was decreased in females and increased in males.Whereas, the abundance of *Acinetobacter* and *Methylobacterium* genera did not showed any change in females post infection with BmCPV; however, the abundance of *Acinetobacter* decreased and *Methylobacterium* increased in males (Fig 2).

### Similarity of bacterial communities in the midgut of BmCPV-infected and healthy control silkworms

A samples distance matrix was calculated by Unifrac software and a heatmap displaying the similarity of bacterial communities in different samples were generated (Fig 3A). Each sample was assigned to one of three clusters: (1) CK-24-M, CK-24-F (the bacterial community of the gut contents at 24 h in the fifth instar male and female larvae, respectively) and CK-144-F (the bacterial community of the gut contents at 144 h in fifth instar female larvae) were grouped into a cluster. (2) CPV-144-F and CPV-144-M (the bacterial community of the gut contents at 144 h of the fifth instar female and male larvae, respectively, post infection with BmCPV) and CK-144-M (the bacterial community of the gut contents at 144 h in fifth instar male larvae) were grouped into a cluster. (3) Other samples were grouped into a cluster, indicating samples were not clustered completely according to sample type (Fig 3A). A similar result was obtained by principal coordinate analysis (PCoA), the samples were clearly separated in the PCoA plot, 51.74%, 33.34% and 6.53% of total variation could be explained by the PC1, PC2 and PC3 axis, respectively (Fig 3B), suggesting the intestinal bacteria community could be affected by gender, development and infection with BmCPV.

**Fig 4:**
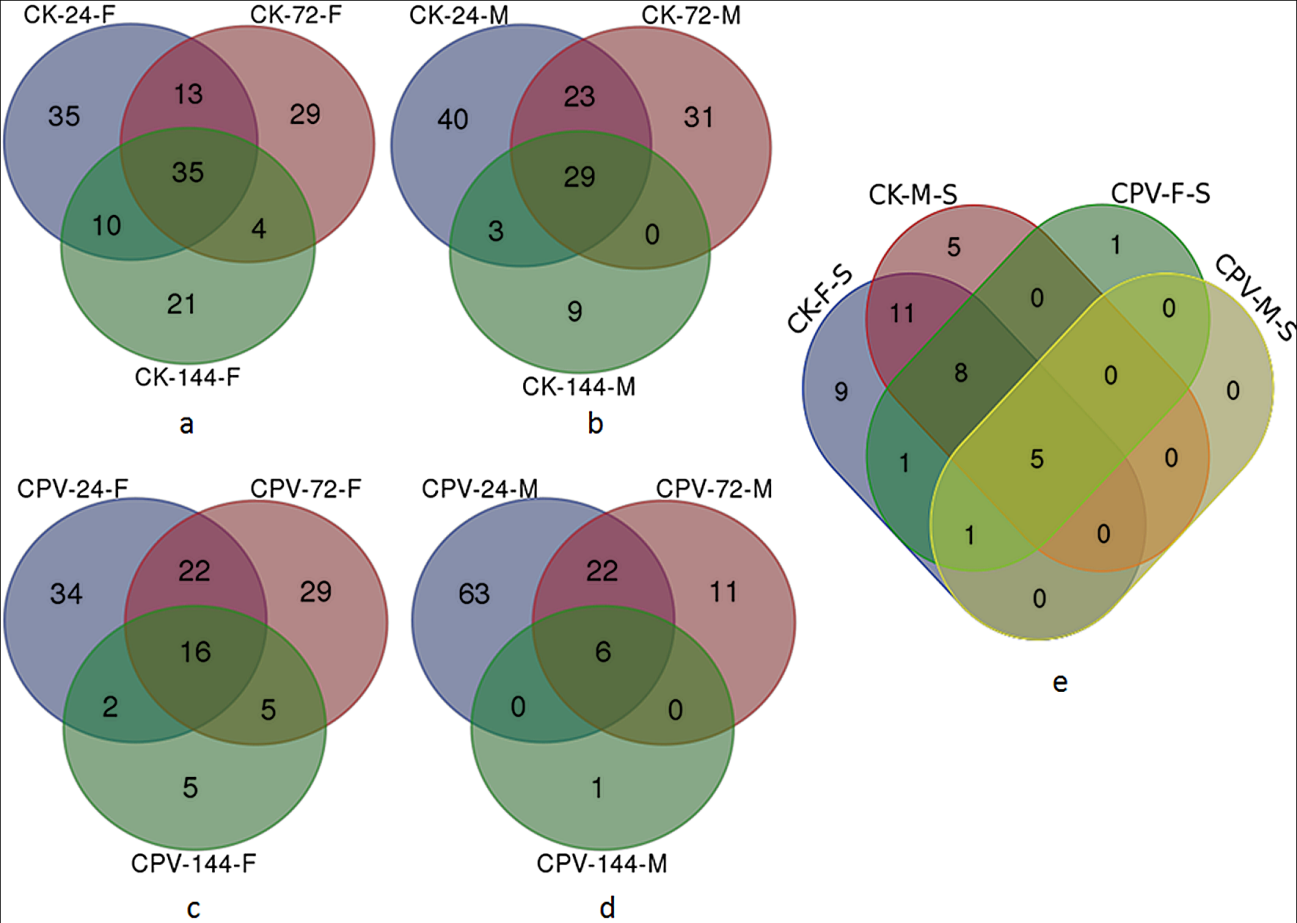
Shared genera analysis of the different samples. Venn diagram showing the unique and shared genera in the different samples. CK (CPV)-24(72,144)-F (M) are samples mentioned in Table 1. CK-F (M)-S was a general designation of CK-24-F (M), CK-72-F (M) and CK-144-F (M). CPV-F (M)-S was a general designation of CPV-24-F (M), CPV-72-F (M) and CPV-144-F (M). (a) for CK-24-F, CK-72-F and CK-144-F samples; (b) for CK-24-M, CK-72-M and CK-144-M samples; (c) for CPV-24-F, CPV-72-F and CPV-144-F samples; (d) for CPV-24-M, CPV-72-M and CPV-144-M samples; (e) for CK-F-S, CK-M-S, CPV-F-S and CPV-M-S samples.

Further to understand changes of bacterial microbiota with gender, developmental stages and the time course of BmCPV infection, Venn diagrams were constructed. Altogether, 35 and 29 genera were found in all samples of the gut contents of healthy female and male larvae, respectively, at different developmental stages of the fifth instar (Fig 4A and 4B) and 24 genera were detected in all larvae (Fig 4E). Post infection with BmCPV, the number of shared genera was reduced; 16 and 6 genera were present in all samples from infected females and males, respectively, (Fig 4C and 4D) and only 6 genera were shared by the female and male larvae (Fig 4E). 15 genera were shared by females and 5 covered by males before and after infection, and only 5 genera (*Enterococcus*, *Delftia*, *Pelomonas*, *Staphylococcus* and *Petrobacter*) were shared by all infected larvae (Fig 4E).

### Phylogenetic tree of predominant genera

**Fig 5:**
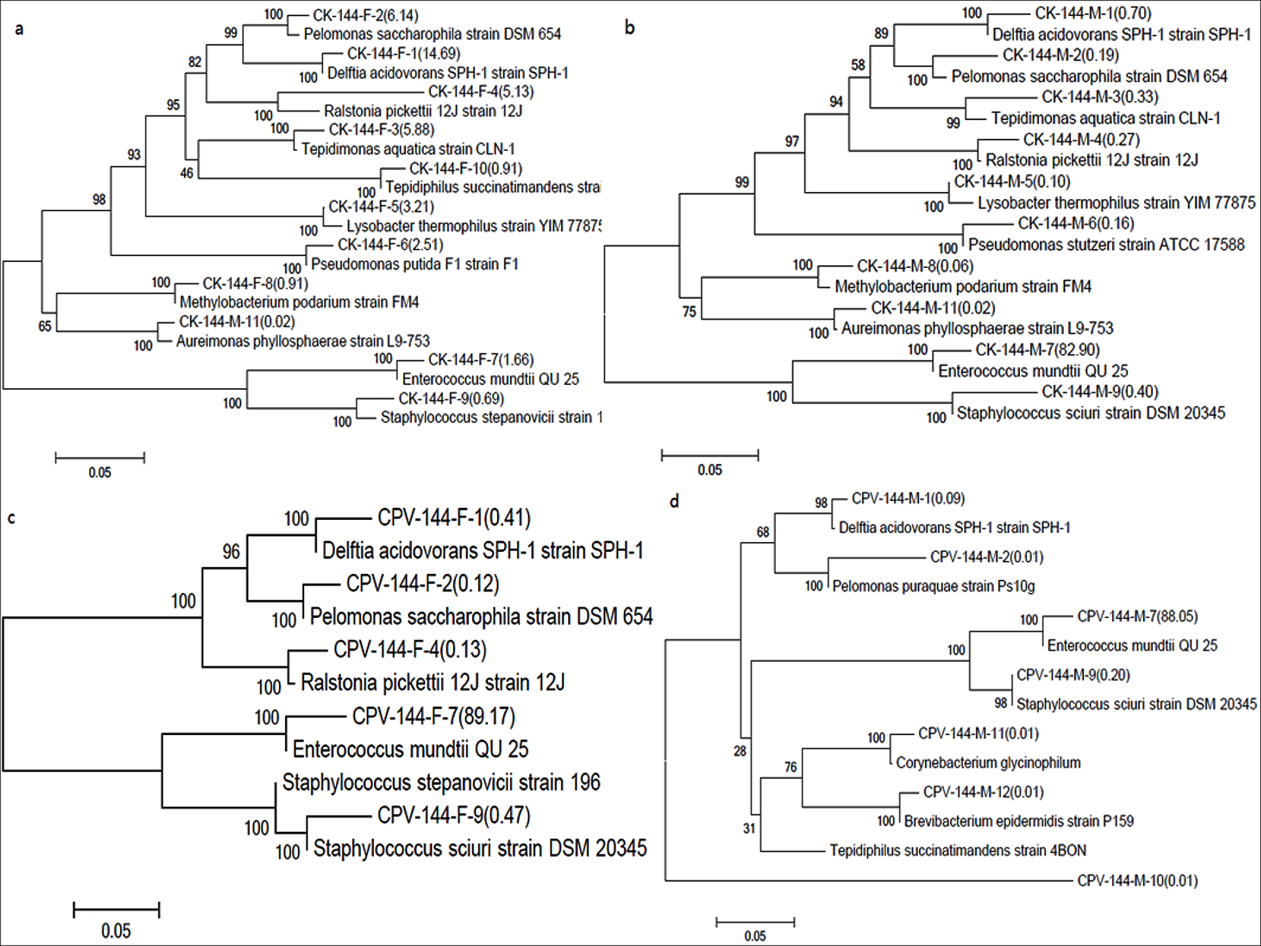
Phylogenetic trees of predominant genera in different samples. The phylogenetic tree was inferred using the neighbor-joining method with MEGA6.0 and the bootstrap value was 1000 replications. Only the branch with bootstrap value >500 are shown in the tree. (a) for CK-144-F, (b) for CK-144-M, (c) for CPV-144-F, and (d) for CPV-144-M; the numbers in parentheses represented the percentage of a predominant genus. Serial numbers from 1–12 represented the *Delftia*, *Pelomonas*, *Tepidimona*, *Ralstonia*, *Aspromonas*, *Pseudomonas*, *Enterococcus*, *Methylobacterium*, *Staphylococcus*, *Tepidimonas*, *Corynebacterium* and *Brevibacterium* genera, respectively. Samples CK (CPV)-144-F (M) were mentioned in Table 1.

To understand the evolutionary relationship of gut predominant bacteria of silkworm, the 16S rRNA gene sequences of predominant genera for CK-144-F, CK-144-M, CPV-144-F and CPV-144-M were selected and performed Blast. Most similar 16S rRNA gene sequences of predominant genera were used to construct the phylogenetic trees. The abundance of a predominant genus was indicated in the phylogenetic tree. The topological structure of the tree for CK-144-F (Fig 5A) was similar to that for CK-144-M (Fig 5B), but the abundance of predominant genera in CK-144-F and CK-144-M was noticeably different. The topological structure and components of the tree for CPV-144-F (Fig 5C) were different compared to CPV-144-M (Fig 5D). The phylogenetic trees of the predominant genera in the control were different compared to the infected silkworms, indicating the composition and abundance of predominant genera were changed following infection with BmCPV. Differences in the change patterns for females and males were also observed (Fig 5).

### Relevance analysis of abundance of the predominant genera

**Fig 6:**
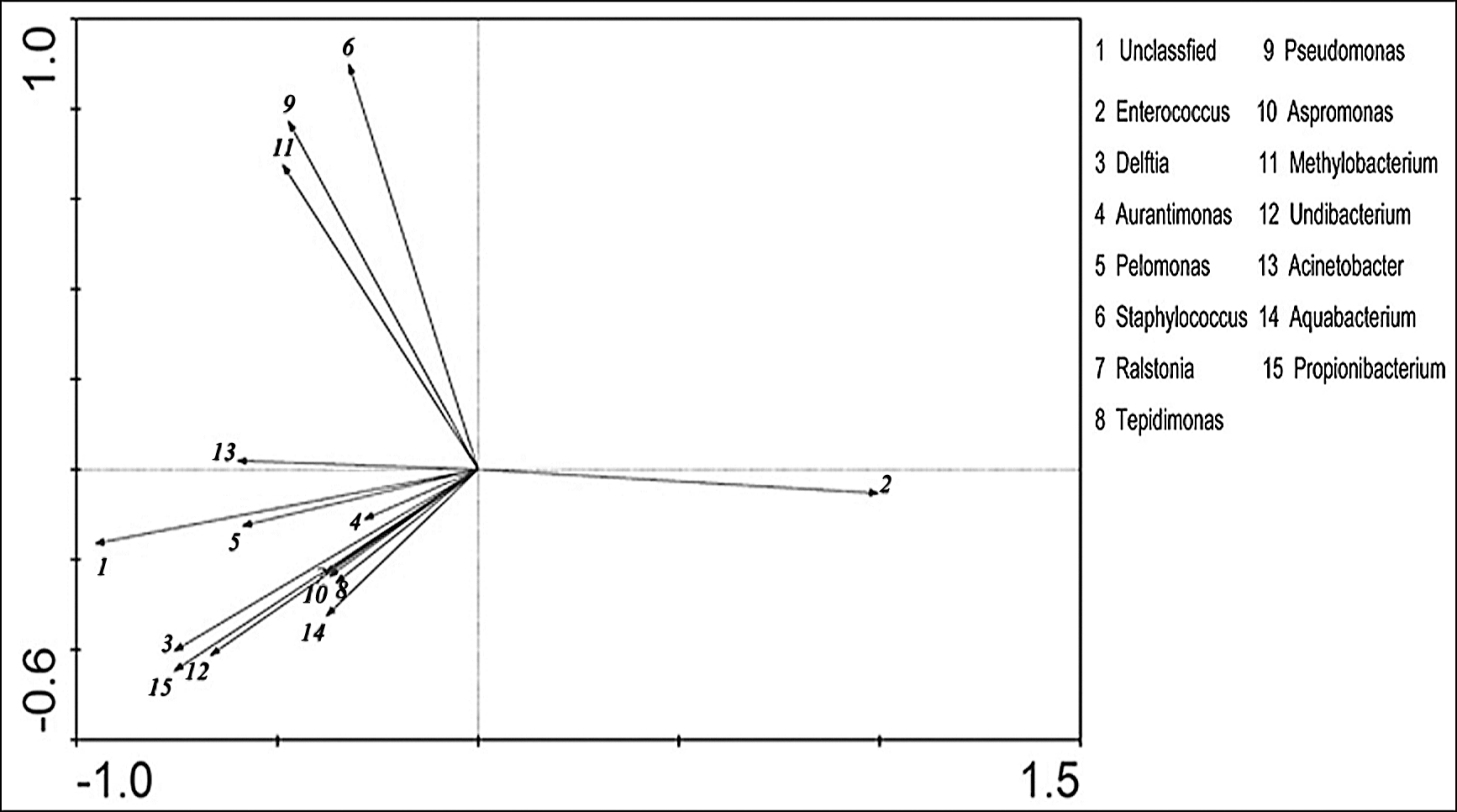
PCA analysis of the predominant genera according to their abundance in the bacterial microbiota. PCA analysis with linear ordination methods was used to explore the correlation between dominant genera using CANOCO 4.5 according to ter Braakand Šmilauer [13].

PCA analysis was used to investigate the relevance of abundance of the 15 predominant genera in the bacterial gut microbiota. These genera were distributed in three different quadrants (Fig 6). There was a positive correlation between the abundance of *Staphylococcus* (6), *Pseudomonas* (9), *Methylobacterium* (11) and *Acinetobacter* (13); and the abundance of other predominant bacteria genera (1, 3–5, 7, 8, 10, 12, 14 and 15) were showed positive correlation with each other. The abundance of *Enterococcus* (2) was correlated negatively with the abundance of the most predominant genera.

## Discussion

Complex intestinal microbial communities were believed to provide some benefits to their host [20]. Human health can be influenced by intestinal microbes [21] and the composition, diversity and functions of intestinal bacteria received a great deal of attention. Insects are a very diverse group and it has been reported that the microbial community can contribute to insect adaptation [22, 23], heat tolerance [24], protection against pathogens or natural enemies [25, 26, 27], reproduction [28] and vector competence [29].

In previous study, the culture-dependent, PCR and 16S rDNA- RFLP (restriction fragment length polymorphism) methods were adopted to investigate the silkworm gut microbiota. Only bacteria of 10–16 genera could be isolated from the gut contents by the culture-dependent method [1], 14 genetypes bacteria were detected by 16S rDNA- RFLP method [30] and 14 genera were found by PCR method [5]. However, compared with the traditional methods, pyrosequencing was applied and 199 genera in the intestinal contents of healthy fifth instar silkworm larvae were indentified, which provided adequate detailed information about silkworm gut microbiota. Rarefaction analysis showed that the sequencing approach was not carried out sufficiently to reach a plateau in this study, indicating the true bacterial diversity in the silkworm gut was underestimated.

Till date, various types of bacteria have been identified in the intestinal contents of insects. The gut bacterial community of the oriental armyworm (*Mythimna separata*) has been investigated; bacteria belongs to *Cyanobacteria*, *Firmicutes*, *Actinobacteria*, *Gracilicutes* and *Proteobacteria* genera were ubiquitous in the gut content [31]. Wild populations of *Aedes albopictus* and *Aedes aegypti* have been shown to harbor principally *Proteobacteria* and *Firmicutes*, including the *Acinetobacter*, *Asaia*, *Delftia*, *Pseudomonas*, *Wolbachia* and *Bacillus* genera, as well as members of the family Enterobacteriaceae [32]. Bacteria of 16 phyla including Proteobacteria and Firmicutes were found in the gut contents of the domesticated silkworm in the present study. Members of the Proteobacteria and Firmicutes phyla are present in armyworms, mosquitoes and silkworms, suggesting the intestinal bacterial microbiota of insects share similar characteristics; however, there are differences in the composition and diversity of bacterial microbiota in different insects, stated that the diversity of gut bacteria in insects can be affected by environment, habitat, diet and developmental stage [33]. Mutualisms between microbes and insects are ubiquitous [34]. The symbiotic relationship between termites and spirochetes was established more than 2000 million years ago [35], the fitness of the bean bug *Riptortus clavatus* can be increased by the symbiotic Protobacteria *Burkholderia* spp., so we speculated the composition and abundance of the predominant gut bacteria in the silkworm is the result of co-adaptation and co-evolution between the silkworm host and intestinal bacteria.

It was reported that the predominant genera in the bacterial microbiota of different silkworm strains were *Brevundimonas*, *Stenotrophomonas*, *Enterobacter* and *Staphylococcus* in strain Dongting × Bibo [30]. *Enterococcus* and *Thermus* were the predominant genera in the C108 and SCN2 strains; however, *Enterobacter* was not found [28]. In the present study, the predominant genera in strain Daizo were *Enterococcus*, *Delftia*, *Ralstonia*, *Pelomonas*, *Tepidimonas*, *Aurantimonas*, *Pseudomonas*, *Aspromonas* and *Staphylococcus*, which showed there was an obvious difference in composition of the intestinal bacteria between different silkworm strains. Previous studies revealed the diversity of locust gut bacteria protects against pathogen invasion [36] and Chromobacterium Csp_P reduces malaria and dengue infection in vector mosquitoes and has entomopathogenic and in vitro anti-pathogen activities [37], so we conjecturing the composition of the intestinal bacterial community might be an important factor resulting in differences of resistance to pathogens between silkworm strains. The pH of the digestive juice, which can be impacted by intestinal bacteria, was involved in the resistance of silkworms to pathogens. *Enterococcus* was a predominant genus in the silkworm gut bacteria. Some species of *Enterococcus* produce acetate and its accumulation might reduce the pH of digestive juice in the grasshopper [38]. *Enterococcus faecalis*, a predominant species of intestinal bacteria in silkworm was commonly found at alkaline pH (8–9) and acidifies its environment through its metabolism [39]. Reducing the pH of the digestive juice can protect an insect from attack by a poisonous parasporal crystal of *Bacillus thuringiensis* or infection with pathogens. The silkworm can be infected only after the virion embedded in the polyhedral bodies of *B*. *mori* nucleopolyhedrovirus (BmNPV) or cypovirus are released at higher pH values. Germination of the microsporum *N*. *bombycis* [40] and activation of *Bacillus thuringiensis* δ-endotoxin required alkaline conditions [41], suggesting the abundance of *Enterococcus* spp. in the bacterial microbiota was involved in the resistance of silkworm to BmNPV, BmCPV and *N*. *bombycis*. Nevertheless, whether metabolic products of the intestinal bacteria can also directly inhibit infection of the silkworm by pathogens deserves further exploration.

Insect intestinal bacteria were involved in digestion and nutrient uptake [42], the composition and diversity of the silkworm intestinal microbiota were showed impact by forage [5]. In the present study, we found the composition and diversity of intestinal microbiota were noticeably reduced at the later period of the fifth instar (144 h of the fifth instar). Food consumption was reduced at the later period of the fifth instar, and the silkworm eventually stopped eating and empties the intestinal content before cocooning, so the decrease of composition and diversity of intestinal microbiota at 144 h of the fifth instar was related to empty the intestinal content for metamorphosis of the silkworm.

Sex differences in the gut microbiome found in the mouse drives hormone-dependent regulation of autoimmunity [43], and sex differences in the immunocompetence and susceptibility to pathogens have been also observed in different insect groups [44, 45]. Both males and females were able to enhance survival in the adult stage as a result of being injected bacteria at the larval stage; it was due to differential gender immune response [46]. According to Ryan evidence, gut symbionts influenced diet selection of male and female *Gryllus pennsylvanicus* differently, and also recommended that sex-specific dietary selection may be because of the fact that male and female crickets have different nutritional requirements [47]. Usually, the resistance to pathogens and feed utilization efficiency for the male silkworms were higher than female silkworm. In our investigation, we found the composition and diversity of gut bacterial microbiota in the silkworm was different between females and males. We speculated the difference of resistance and feed utilization efficiency between genders could be a result of differences in the gut microbiome.

The resistance of insects to pathogens is influenced by intestinal bacteria; conversely, the composition and diversity of intestinal bacteria are influenced by infection with pathogen. Silkworm cytoplasmic polyhedrosis is usually associated with bacterial gut disease. In the current study, we found bacterial diversity in the gut content was decreased after infection with BmCPV and the abundance of bacteria changed noticeably with the course of BmCPV infection. The abundance of *Enterococcus* spp. at 144 h post infection increased to 88.75%, which was 1.28-fold more than control and the abundance of *Pseudomonas* spp. decreased to 0.02%, which was 27-fold lower compared to untreated batch. Our finding suggested that homeostasis of the intestinal microbiota might be broken by infection with BmCPV, which initiated generation of bacterial gut disease of the silkworm. Some *Enterococcus* spp. were opportunistic pathogens of silkworm [48, 49], the number of bacteria of the genus *Enterococci* was increased in silkworms infected with *N*. *bombycis*[6], a similar result was observed in this study, which suggested that the increase of *Enterococcus* spp related to the immune response in the silkworm.

A recent investigation revealed an antibacterial peptide cecropins gene expression level in silkworm was upregulated after infection with BmCPV [50]. Gram-negative bacteria were generally more sensitive to cecropins than Gram-positive organism [51]. In the present study, abundances of the predominant genera *Enterococcus* and *Staphylococcus* belonging to Gram-positive bacteria were roughly increased and copiousness of the predominant genera belonging to Gram-negative bacteria was decreased after infection with BmCPV infection. Therefore, it is suggested that observed changes in relative abundance was related to the upregulation of cecropins after infection with BmCPV.

Co-existence and competition relationship between gut bacteria were found in the silkworm. The abundance of *Staphylococcus*, *Pseudomonas*, *Methylobacterium* and *Acinetobacter* were in positive correlation with each other, while the abundance of *Enterococcus* spp. was in negative correlation with the most predominant genera, implying that resistance of the silkworm to infection with pathogens can be increased by the use of probiotics and optimization of the gut bacterial microbiota.

Some species of *Enterococcus* were probiotic and beneficial to healthy of host. *Enterococcus faecalis* CECT7121 is a probiotic strain that has been demonstrated to implant itself, persist and induce protective immune responses in several biological models [52,53,54]. Use the liquid probiotic form *Enterococcus faecalis L3* in infants had a positive impact on overall health and can increase resistance to acute respiratory infections [55]. In insect, previous studies indicated that intestinal bacteria inhibit the growth of *Bacillus thuringiensis* in the larvae of the oriental tea tortrix, *Homona magnanima* [56] and the substance secreted by *Enterococcus* inhibit the germination of *N*. *bombycis* spores [57], and furthermore, genus *Enterococcus* is the most dominant bacteria in the silkworm intestinal microflora, therefore, we speculated that *Enterococcus* can be used as probiotics to defense against pathogen invasion.

## Supporting Information

S1 Table Proportion of genera in the intestinal bacterial community at different time points in the fifth instar of healthy and BmCPV-infected silkworms. CPV were genera detected after infection with BmCPV. 24, 72 and 144 represent the gut contents were respectively collected at 24, 72 and 144 h in the fifth instar. The original data of pyrosequencing related to this article can be found in GenBank.
(DOCX)

S2 Table Accession numbers of original data. CK (CPV)-24(72,144)-F (M) are samples mentioned in Table 1.
(DOCX)
