# Supplementary material for: Effects of BmCPV Infection on Silkworm Bombyx mori Intestinal Bacteria
Source: PLoS One. 2016 Jan 8;11(1):e0146313. doi: 10.1371/journal.pone.0146313 (PMC4706323; doi:10.1371/journal.pone.0146313)
Supplement: S1 Table — CPV were genera detected after infection with BmCPV. 24, 72 and 144 represent the gut contents were respectively collected at 24, 72 and 144 h in the fifth instar. The original data of pyrosequencing related to this article can be found in GenBank. (DOCX) [file pone.0146313.s001.docx]

**Effects of BmCPV Infection on** **Silkworm *Bombyx mori* Intestinal Bacteria**

Zhenli Sun^1^*, Yahong Lu^1^*, Hao Zhang^1^, Dhiraj Kumar^1^, Bo Liu^1^, Yongchang Gong^1^, Min Zhu^1^, Liyuan Zhu^1^, Zi Liang^1^, Sulan Kuang^1^, Fei Chen^1^, Xiaolong Hu^1,2^, Guangli Cao^1,2^, Renyu Xue^1,2^ , Chengliang Gong^#^ ^1,2^

**S1 Table** **Proportion of genera in the intestinal bacterial community at different time points in the fifth instar of healthy and BmCPV-infected silkworms**

| Genera | 24 | 72 | 144 |
| --- | --- | --- | --- |
| Enterococcus | 26.09% | 2.69% | 68.98% |
| Enterococcus-CPV | 0.27% | 4.38% | 88.75% |
| Delftia | 8.82% | 16.79% | 3.12% |
| Delftia-CPV | 5.43% | 12.14 | 0.29 |
| Pelomonas | 5.62% | 3.01% | 1.22% |
| Pelomonas-CPV | 2.16% | 3.17% | 0.08% |
| Aurantimonas | 23.30% | 4.14% | 0.09% |
| Aurantimonas-CPV | 1.30% | 0.12% | 0% |
| Ralstonia | 3.77% | 2.23% | 1.11% |
| Ralstonia-CPV | 0.31% | 0,71% | 0.08% |
| Tepidimonas | 2.77% | 1.69% | 1.29% |
| Tepidimonas-CPV | 0.16% | 0.16% | 0.05% |
| Pseudomonas | 2.52% | 1.83% | 0.56% |
| Pseudomonas-CPV | 8.74% | 2.42% | 0.02% |
| Aspromonas | 2.60% | 1.26% | 0.64% |
| Aspromonas-CPV | 0.05% | 0.31% | 0.01% |
| Staphylococcus | 1.85% | 0.64% | 0.45% |
| Staphylococcus-CPV | 17.16% | 2.42% | 0.37% |
| Aquabacterium | 0.63% | 0.39% | 0.15% |
| Aquabacterium-CPV | 0.01% | 0.12% | 0.01% |
| Methylobacterium | 0.73% | 0.78% | 0.20% |
| Methylobacterium-CPV | 4.93% | 0.90% | 0.02% |
| Acinetobacter | 0.66% | 0.69% | 0.09% |
| Acinetobacter-CPV | 1.04% | 1.21% | 0.06% |
| Undibacterium | 0.52% | 0.67% | 0.08% |
| Undibacterium-CPV | 0.18% | 0.43% | 0.01% |
| Propionibacterium | 0.51% | 0.57% | 0.11% |
| Propionibacterium-CPV | 0.22% | 0.96% | 0 |

–CPV were genera detected after infection with BmCPV. 24, 72 and 144 represent the gut contents were respectively collected at 24, 72 and 144 h in the fifth instar
