# Supplementary material for: Effects of BmCPV Infection on Silkworm Bombyx mori Intestinal Bacteria
Source: PLoS One. 2016 Jan 8;11(1):e0146313. doi: 10.1371/journal.pone.0146313 (PMC4706323; doi:10.1371/journal.pone.0146313)
Supplement: S2 Table — CK (CPV)-24(72,144)-F (M) are samples mentioned in Table 1. (DOCX) [file pone.0146313.s002.docx]

**Effects of BmCPV Infection on** **Silkworm *Bombyx mori* Intestinal Bacteria**

Zhenli Sun^1^*, Yahong Lu^1^*, Hao Zhang^1^, Dhiraj Kumar^1^, Bo Liu^1^, Yongchang Gong^1^, Min Zhu^1^, Liyuan Zhu^1^, Zi Liang^1^, Sulan Kuang^1^, Fei Chen^1^, Xiaolong Hu^1,2^, Guangli Cao^1,2^, Renyu Xue^1,2^ , Chengliang Gong^#^ ^1,2^

**S2 Table The accession number of original data**

| sample | Accession number |
| --- | --- |
| CK-24-M | SRR2054752 |
| CK-24-F | SRR2054751 |
| CK-72-M | SRR2054754 |
| CK-72-F | SRR2054753 |
| CK-144-M | SRR2054750 |
| CK-144-F | SRR2054749 |
| CPV-24-M | SRR2054759 |
| CPV-24-F | SRR2054758 |
| CPV-72-M | SRR2054761 |
| CPV-72-F | SRR2054760 |
| CPV-144-M | SRR2054757 |
| CPV-144-F | SRR2054756 |

CK (CPV)-24(72,144)-F (M) are samples mentioned in Table 1
